# Distribution Patterns of Grasshoppers and Their Kin over the Eurasian Steppes

**DOI:** 10.3390/insects12010077

**Published:** 2021-01-15

**Authors:** Michael G. Sergeev

**Affiliations:** 1 Department of General Biology and Ecology, Novosibirsk State University, 2, Pirogova Street, 630090 Novosibirsk, Russia; mgs@fen.nsu.ru; 2 Laboratory of Invertebrate Ecology, Institute of Systematics and Ecology of Animals, Siberian Branch, Russian Academy of Sciences, 11, Frunze Street, 630091 Novosibirsk, Russia

**Keywords:** Orthoptera, biological diversity, fauna, distribution, range, population, pest, rare species

## Abstract

**Simple Summary:**

The steppe zone is the huge area in temperate Eurasia where different grasslands form the main type of vegetation. The main part of this life zone has been transformed by human activity and used for crop cultivation (mainly wheat) and livestock. Regional and local transformations and/or climate changes can result in some shifts in species and population distribution of Orthoptera. Over this area, the climatic conditions and the dominated grasslands are suitable for numerous grasshoppers and their relatives, especially the graminivorous ones. The general level of their diversity is usually high. Many species are very abundant and may become important pests. However, there are also many rare species. The main aims of this paper are to reveal general patterns of Orthoptera distribution in the Eurasian steppes, to evaluate long-term trends of changes in distribution of taxa and populations, and to estimate the potential for population changes relative to human activity and global warming trends.

**Abstract:**

The main aims of this paper are to reveal general patterns of Orthoptera distribution in the Eurasian steppes, to evaluate long-term trends of changes in distribution of taxa and populations, and to estimate the potential for population changes relative to human activity and global warming trends. The main publications concerning diversity and distribution of these insects over the steppes are analyzed. The fauna of the Eurasian steppes includes more than 440 species of Orthoptera. The general distribution of grasshoppers and their kin in the Eurasian steppes reflects their common associations with different grasslands. The species richness increases from the relatively cold forest-steppes to the semi-deserts with their warm summer. There are some endemic or subendemic taxa, including the tribe Onconotini (Tettigoniidae). The populations’ distribution of Orthoptera is also analyzed. The populations of native Orthoptera extend through all the herbaceous landscapes. Under these conditions, the interrelating of colonies of each species may result in great abundance. The population distribution of three species locusts (*Locusta migratoria*, *Calliptamus italicus*, *Dociostaurus maroccanus*) is also discussed. Some notable changes of their populations’ distribution and dynamics are characterized. The situation with rare Orthoptera is estimated. Retrospective and prospective of the steppe fauna of Orthoptera are discussed.

## 1. Introduction

**Figure 1:**
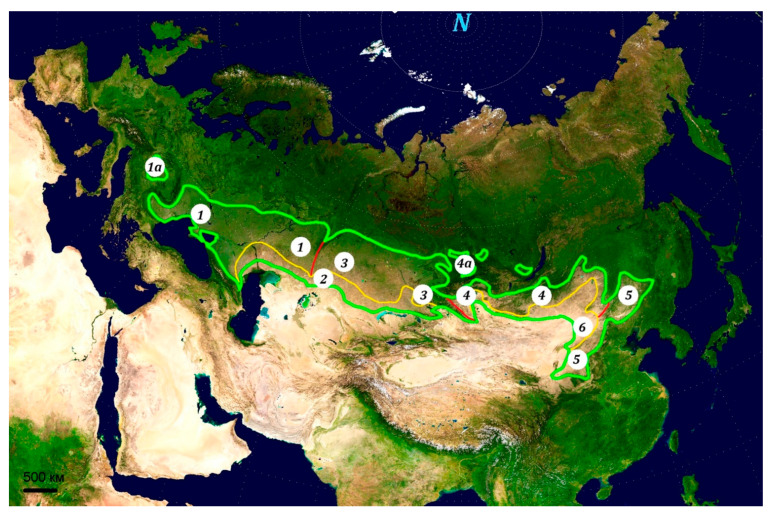
Distribution of the Eurasian steppes and their subregions. The boundaries of the steppe and the semi-desert life zones based on [1,2,3,4] with some changes and simplification. Boundaries: green—general outline of the Eurasian steppes including the semi-deserts and excluding the mountain steppes of South-West and Central Asia; yellow—boundary between the dry steppes and the semi-deserts; red—boundaries between western, central, and eastern parts of the steppes. *1*—European (western) parts of Scythian biogeographic subregion; *1a*—Pannonian Plain; *2*—Kazakhstan Province of Saharan-Gobian subregion; *3*—West-Siberian parts of the Scythian subregion; *4*—Mongolian-Siberian parts of Scythian subregion; *4a*—insular parts of forest-steppes and steppes of South Siberia; *5*—Dongbei Province of Scythian subregion; *6*—Mongolian Province of Saharan-Gobian subregion. The basic map is “A Two Point Equidistant Projection of Asia” (the control points are at 35° N 40° E and 35° N 140° E; the reticle is 10 degrees in latitude and longitude, with the central meridian at 90° E); the source image is a product of NASA’s Blue Marble Project public domain.

The steppe zone is the huge area in temperate Eurasia where different grasslands form the main type of vegetation [1,2,3,4] (Figure 1). The adjacent forest-steppes and semi-deserts should be included in this area [5,6]. Average temperatures are relatively low (mean temperatures of the warmest month vary from 16 °C to 25 °C, the same for the coldest month from −1 °C to −34 °C), and annual precipitation amounts are relatively low (for the temperate regions) and vary from 115 mm in the inner parts of Eurasia up to 640 mm in the westernmost and the easternmost parts of the Eurasian steppes [3]. The main part of this life zone has been transformed by human activity and used for crop cultivation (mainly wheat) and livestock. The steppes were commonly used as pasturelands for many centuries. However, the European steppes were gradually transformed to agriculture fields several centuries ago. In the beginning of the 20th century, some territories were also ploughed in South Siberia. Later, in the middle of the last century, during the so-called Virgin Land campaign, huge steppe areas were ploughed, and many of the remaining areas of steppe have become overgrazed. As a result, many habitats of grasshoppers and their relatives have been destroyed or damaged. Such regional and local transformations and/or climate changes can result in some shifts in species and population distribution.

From the zoogeographic point of view, the steppe zone almost coincides with the Scythian (or Euro-Siberian Steppe) subregion of Palaearctic erected mainly on the basis of the species distribution analysis [7,8,9,10,11]. Over this area, climatic conditions and dominated grasslands are suitable for numerous grasshoppers and their relatives, especially the graminivorous species. The general level of their diversity is usually high [8,9,10,11,12,13]. Many species are very abundant and may become important pests. However, there are also many rare species. Besides, numerous steppe or steppe-like habitats are distributed over the mountains of arid and semi-arid areas of temperate and subtropical Eurasia from South-East Europe to the Hengduan mountains. These mountains are characterized by a rather complicated relief and high variability of local climatic conditions that commonly result in high levels of the local diversity of Orthoptera [9,10,11]. Certainly, there are also several very abundant grasshopper species that may be some potential pests.

The main aims of this paper are to reveal general patterns of Orthoptera distribution in the Eurasian steppes, to evaluate long-term trends of changes in distribution of taxa and populations, and to estimate prospective of their population changes relative to human activity and global warming trends.

## 2. Methods and Materials

Both qualitative and quantitative data were used. The analysis of geographic distribution was based on published and unpublished species range maps. Species data points for Eurasian Orthoptera were plotted onto base maps, usually on a scale of 1:25,000,000. My own collections, the collections of different museums, and published data were used [8,9].

The analysis of geographic and ecological distribution was based on quantitative samples collected in natural, semi-natural, and human-dominated habitats. Samples captured during a fixed period of time were made in every habitat investigated [8,14]. Using this method, insects were caught with a standard net over a period of 10–30 min. Results for every habitat were recalculated for an hour. This method allows us to obtain repeatable and comparable results for different regions and years. In some cases, hand collecting and sweep nets were also used to get additional information. In many habitats, orthopteran densities were also counted on 25–100 arbitrarily placed square plots 0.25 × 0.25 m^2^ (sometimes 0.5 × 0.5 m^2^ or 1 × 1 m^2^). After that, the average density was estimated for every habitat investigated. The samples were collected in the Eurasian steppe by the expeditions of Department of General Biology and Ecology (Novosibirsk State University) and the Laboratory of Insect Ecology (now Laboratory of Invertebrate Ecology, Institute of Systematics and Ecology of Animals, Siberian Branch, Russian Academy of Sciences) from 1972 to 2020. Numerous published papers and books (see the next parts of the publication) describing orthopteran distribution in different parts of the steppe life zone were also analyzed. In almost all cases, I use modern names of taxa [15] without special comments and exclude evident synonyms and misidentifications published in some papers and books.

## 3. History of Species Descriptions and Distributions

The first data concerning the steppe Orthoptera were published in the 18th century by P. S. Pallas [16,17]. He described several new taxa, e.g., *Gryllus (Locusta) barabensis* (now *Angaracris barabensis*), *G. muricatus* (*Asiotmethis muricatus*), *G. miniatus* (*Oedipoda miniata*), *G. variabilis* (*Celes variabilis*), *Gryllus (Tettigonia) pedo* (*Saga pedo*), *G. laxmanni* (*Onconotus laxmanni*), *Gryllus (Acheta) desertus* (*Melanogrylus desertus*), and *Gryllus (Acrida) oxycephalus* (*Acrida oxycephala*). In the middle of the 19th century, G. Fischer von Waldheim [18] listed more than 50 species of Orthoptera associated with the steppe territories. Later, E. Eversmann [19] included more than 80 species (except the evident synonyms and some typical desert forms) in his relatively comprehensive list of grasshoppers and their kin of the so-called Volga-Ural region (Tettigoniidae—18 species, Gryllidae—5, Gryllotalpidae—1, Tridactylidae—1, Tetrigidae—2, Pamphagidae—1, and Acrididae > 54 species).

In 1890–1920s, several entomologists started to collect and analyze the orthopteran faunas of the different parts of the steppes. For instance, B. P. Uvarov [20,21] and D. P. Dovnar-Zapolsky [22] characterized Orthoptera distribution over the North Caucasus. S. I. Medvedev [23] described diversity of Orthoptera in the Nature Reserve Askania-Nova (now the biosphere reserve) and its vicinities in the dry steppe area of Ukraine. N. N. Adelung [24,25], N. N. Umnov [26], S. P. Tarbinskij [27], and W. Wnukowskij [28] listed the Orthoptera taxa of several regions of North Kazakhstan and West Siberia. E. F. Miram [29] and G. Ja. Bey-Bienko [30] published some data about orthopteran distribution over the mountains of South Siberia. Uvarov [31] and E. Pylnov [32] described diversity of Orthoptera in the Transbaikal Region (Dauria) and adjacent Mongolia. Bey-Bienko [33] also listed some species from the easternmost steppe territories in North-East China.

In 1905, G. G. Jacobson [34] summarized all known data on taxonomy and distribution of Orthoptera from the former Russian Empire and adjacent countries and published keys and annotated lists of taxa. He listed more than 158 species distributed in the steppe part of the Empire (Tettigoniidae—about 60 species, Gryllidae—7, Myrmecophilidae—1, Mogoplistidae—1, Gryllotalpidae—2, Tridactylidae—3, Tetrigidae—3, Pamphagidae—2, and Acrididae—about 80 species). The field guide “Grasshoppers of the European part of the USSR and West Siberia” was published in 1925 [35]. In this book, Uvarov characterized main morphological and ecological peculiarities of local grasshoppers and described their distribution patterns. He listed 83 species of Acridoidea (Tetrigidae—3, Pamphagidae—2, Acrididae—78) associated with forest-steppe, steppe, and semi-desert life zones.

In the 1950s, Bey-Bienko and L. L. Mistshenko published several books concerning some taxa of Orthoptera of the former USSR and adjacent territories: Acridoidea and Tetrigidae [36], Catantopinae *s. l.* [37], and Phaneropterinae [38]. They revised many groups and described many new taxa and the distribution of all known species and subspecies. They also analyzed the general distribution patterns and the main ecological peculiarities of noted groups. Later, R. P. Berezhkov [39] published the comprehensive book about Acridoidea and Tetrigidae of West Siberia.

Bey-Bienko [12] summarized all data on orthopteran distribution over the steppe life zone (mainly for the territory of the former USSR). He noted the relatively high level of species richness in the steppes per se (about 150 species), the moderate level in the semi-deserts (about 120), and the relatively low diversity in the forest-steppes (about 90 species). Bey-Bienko also discussed high abundance and dominance of grasshoppers and emphasized that *Glyptobothrus dubius* (Zubovsky) might be the true indicator of the Eurasian steppes. Besides, he mentioned several widely distributed species of other genera (e.g., *Stenobothrus nigromaculatus* (Herrich-Schäffer), *Stenobothrus fischeri* (Eversmann), *Stenobothrus eurasius* Zubovsky, *Euchorthippus pulvinatus* (Fischer de Waldheim), *Montana eversmanni* (Kittary), *Montana striata* (Kittary), etc.) also preferring steppe habitats. Almost all these species are associated with grasses and may be qualified as graminicoles (sensu Uvarov [13]). Besides, there are many herbicoles and some arboricoles (especially some bush-crickets and grasshoppers of the subfamily Melanoplinae in the northern part of the region) and a number of terricoles (mainly from the family Pamphagidae and the subfamily Locustinae (=Oedipodinae) in the dry steppes and the semi-deserts). In the steppes, almost all species of Orthoptera belong to two families, namely Tettigoniidae and Acrididae. There are also a few Gryllidae, Myrmecophilidae, Mogoplistidae, Gryllotalpidae, Tridactylidae, Tetrigidae, Pyrgomorphidae, and Pamphagidae.

In his initial analysis of the geographical regions of the Eurasian steppes (subject to further elaboration later, see also Figure 1), Bey-Bienko proposed that they may be split into three faunistic subregions: (1) Pontic (or Pontic-Caspian), (2) West-Asian, and (3) Siberian-Mongolian. He noted that there were a few species distributed over all steppes. The first Subregion occupies the western part of the Eurasian steppe, up to the Ural mountains. There are some endemics and subendemics, mainly from the genera *Poecilimon* Fischer and *Isophya* Brunner von Wattenwyl, *Bradyporus multituberculatus* (Fischer de Waldheim), *Montana medvedevi* (Miram), *Gryllomorpha miramae* S. Medvedev, *Asiotmethis tauricus* (Serg. Tarbinsky), etc. The orthopteran fauna of the subregion is evidently associated with Mediterranean and European faunas [12,21]. There are many species distributed over the western parts of Palaearctic. Besides, some forms associated with the deserts also penetrate the southern part of the Pontic subregion. Bey-Bienko also emphasized that there are many species preferring forbs, bushes, and trees (herbicoles and arboricoles).

The West-Asian subregion occupies territories between the Ural Mountains and the north-western parts of the Altay-Sayan Mountains. It is characterized by a few endemics and subendemics (*Asiotmethis jubatus* (Uvarov), *Mesasippus arenosus* (Bey-Bienko), etc.). The regional fauna includes both some boreal species (*Bohemanella frigida* (Boheman)), forms associated with semi-deserts and deserts (*Dociostaurus kraussi* Ingenitzky, *Sphingonotus coerulipes* Uvarov, *Eremippus simplex* (Eversmann)), and species mainly distributed in the eastern parts of Palaeartic (*Celes skalozubovi* Adelung, *Gampsocleis sedakovii* (Fischer de Waldheim)). There are several species common for the Pontic and the West-Asian steppe subregions, namely *Saga pedo* (Pallas), *Onconotus* spp., *Miramiola pusilla* (Miram), *Montana striata,* etc. A few arboricoles and herbicoles occur in this area. The Siberian-Mongolian subregion is characterized by some endemics and subendemics: *Deracanthella aranea* (Fischer de Waldheim), *Montana tomini* (Pylnov), *Uvarovina venosa* (Fischer de Waldheim), *Dasyhippus barbipes* (Fischer de Waldheim), *Chorthippus hammarstroemi* (Miram), etc. There are many species mainly distributed either in East Palaearctic (*Ognevia longipennis* (Shiraki), *Prumna primnoa* (Motschulsky), *Megaulacobothrus aethalinus* (Zubovsky), *Stethophyma magister* (Rehn), *Schmidtiacris schmidti* (Ikonnikov), etc.) or in the steppes and the semi-deserts of Mongolia (especially from the tribe Bryodemini). The local fauna includes many terricoles and graminicoles, however, there are also some herbicoles and arboricoles (mainly from the subfamily Melanoplinae) in the mountains of South Siberia and Mongolia.

Later, in the second half of the 20th century and in the beginning of the 21st century, the faunas of some part of the Eurasian steppes were catalogued and analyzed (several European steppe regions [40,41,42,43], Kazakhstan [44,45,46], Tuva (in the central part of the mountains of South Siberia) [47,48,49], Mongolia [50,51,52], and North China [53,54,55,56,57]). M. G. Sergeev [8,10,11] published lists of Orthoptera for main biographical regions of the Asian steppes. S. Yu. Storozhenko [58] revised taxonomic diversity of the long-horned orthopterans of the Asian part of Russia. This means, nowadays, we have more comprehensive data on orthopteran distribution and diversity in the Eurasian steppes. However, some unsolved taxonomical and phylogenetic problems remain unclear despite some opportunities of an acoustic analysis and molecular approaches (e.g., with the members of the genus *Glyptobothrus* Chopard) (see [11,47,48,49,59]. Additionally, of course, there are new taxa that remain to be found and described, especially in the eastern part of the steppes. Even so, we do have a relatively comprehensive data set that allows for a fairly accurate analysis of geographic distributions.

## 4. Geographic Distribution of Taxa and Diversity

The fauna of the Eurasian steppes (without the mountain steppes of South-West Asia, Central Asia, and Tibet) includes more than 440 species of Orthoptera; about half of them are the members of the family Acrididae, and about 30% are bush-crickets of the family Tettigoniidae. The general distribution of grasshoppers and their kin in the Eurasian steppes reflects the southern thermophilic character of these insects and their common associations with different grasslands, such as meadows, steppes, and semi-deserts. The species richness increases from the relatively cold forest-steppes to the semi-deserts with their warm summer (Table 1). However, the fauna of the Mongolian semi-deserts includes fewer species than the fauna of the South Siberian and the Mongolian steppes.

More than 240 species occur in the European steppes (including the local forest-steppes and the semi-deserts). There are many Tettigoniidae (up to 38–39%). Among them are several genera and species of the tribe Pholidopterini and many endemic and subendemic forms from the tribe Odonturini (especially from the genera *Isophya* and *Poecilimon*). Some species of the subfamilies Bradyporinae and Meconematinae are also here. The regional fauna of grasshoppers includes a number of Melanoplinae. There is also at least one species of the subfamily Pezotettiginae. However, only a few grasshoppers of the family Pamphagidae occur here. *Bradyporus multituberculatus*, *Isophya stepposa* Bey-Bienko, *I. doneciana* Bey-Bienko, *Poecilimon ukrainicus* Bey-Bienko, and *Montana medvedevi* are among more or less typical endemics or subendemics.

The orthopteran fauna of the West-Asian steppes is less diverse and includes about 160 species. The only small tribe, Glyphonotini, characterizes this fauna (actually its southern part). *Asiotmethis zacharjini* (Bey-Bienko), *Eclipophleps beybienkoi* Maljkovsky, *Eclipophleps kazacha* Maljkovsky, *Aeropedellus baliolus* Mistshenko, and several species of *Mesasippus* Serg. Tarbinsky are local endemics or subendemics.

The eastern parts of the Eurasian steppes are more diverse again. Their fauna includes about 215 species. Among them are several genera and species of bush-crickets from the tribes Drymadusini and Bergiolini, with at least 16 species from different genera of Pamphagidae (including several forms from the tribe Haplotropidini). Besides, some grasshoppers of the tribes Hypernephiini and Bryodemini occur here. *Deracanthina deracanthoides* (Bey-Bienko), *Zichya baranovi* (Bey-Bienko), *Bienkoxenus beybienkoi* (Stebaev), *Montana tomini*, several forms from the genera *Eotmethis* Bey-Bienko, *Filchnerella* Karny (Pamphagidae), *Eclipophleps* Serg. Tarbinsky, *Glyptobothrus*, and *Chorthippus* Fieber are either endemics or subendemics of the region.

Many species are widely distributed across almost all Eurasia, usually from the Atlantic Ocean to the Pacific, however, the ranges of some species include the wide set of the life zone in the central part of Eurasia, commonly from the semi-deserts to the boreal forests, but can be limited by the boreal areas and some mountains in the western and the eastern parts of the continent [8,60]. Among these widely distributed forms are *Podisma pedestris* (Linnaeus), *Gomphocerus sibiricus* (Linnaeus), *Aeropedellus variegatus* (Fischer de Waldheim), *Stethophyma grossum* (Linnaeus), *Bryodemella tuberculata* (Fabricius), *Chrysochraon dispar* (Germar), *Omocestus haemorrhodalis* (Charpentier.), *Omocestus viridulus* (Linnaeus), *Decticus verrucivorus* (Linnaeus), *Epacromius pulverulentus* (Fischer de Waldheim), and *Oedaleus decorus* (Germar). Several widely distributed grasshoppers (e.g., *Gomphocerus sibiricus*, *Bohemanella frigida*, *Podisma pedestris*) have isolated populations in the mountains of south temperate Eurasia (from Pyrenees to Central Asia).

Bey-Bienko [12] tried to estimate biogeographic significance of meridional (inter-sectoral) geographic boundaries only in the steppes mainly related to mountain ranges. However, the inter-zonal boundaries (i.e., the boundaries between the life zones and the subzones) can be also very important. Stebaev and Sergeev [61] compared significance of the inter-zonal and the meridional boundaries relative to distribution of Siberian Orthoptera range borders and showed that the inter-zonal boundaries may be more important than the meridional ones. In many cases, the notable roles of the inter-zonal boundaries are determined by the northern range borders. For instance, the range borders of many species associated with deserts and semi-deserts do not cross the northern limits of the steppe per se or the northern boundaries of the dry steppe [8,61]. Low temperatures predominate in restricting the northward distribution of species [9]. Intensification of continental climate mainly limits the spreading from the west and from the east to the center of Eurasia. This allowed the suggestion of some general schemes of regionalization of the Palaearctic Region and its subregions, including forest-steppe, steppe, and semi-desert ones [8,9,10,11].

The Scythian (or Steppe—according to Uvarov [21]) subregion covers the areas of forest-steppes and steppes from East Europe to North China [8,9,10,11] (Figure 1). The majority of orthopteran subfamilies and tribes are mainly associated with tropical and subtropical territories. Only a few subfamilies (Zichyinae, Thrinchinae) and tribes (Odonturini, Drymadusini, Bergiolini, Gampsocleidini, Onconotini, Chrysochraontini, Hypernephiini et al.) are mainly associated with the Palaearctic Region. The subregion includes at least several provinces:

(1) The Russian-Siberian Province mainly consists of forest-steppes and deciduous forests of Europe and South Siberia. Distribution of the range boundaries (mainly associated with Manchurian and Mediterranean species) reveals the existence of several districts (Oka-Baikal, Dnepr-Argun, Sungary, and Pannonian (?—M.S.)). The Pannonian Plain may be its westernmost and isolated part (Figure 1(*1a*)). There is at least one endemic, namely *Isophya costata* Brunner von Wattenwyl. Several insular areas are also in South Siberia (Figure 1(*4a*)). There is also one common endemic species—*Aeropedellus reuteri* (Miram).
(2) The Sarmathian Province occupies the steppe life zone per se, from South-East Europe to East Mongolia, including the main parts of the mountains of South Siberia and North Mongolia. The Province is characterized by many endemics and subendemics, including the mountain ones. It may be divided into several subprovinces (from west to east: Pontic, West-Siberian, Altay-Sayan, Daurian). The Pontic Subprovince is characterized by numerous species commonly associated with the Mediterranean region [21,22]. The West-Siberian Subprovince is distinguished by the penetration of deserticolous Turanian Orthoptera. The Gobian forms are found in the Altay-Sayan Subprovince. The Daurian Subprovince is characterized by colonization by Manchurian Orthoptera.
(3) The Dongbei Province includes the eastern part of the subregion (Figure 1(*5*)) and is connected with the southward deviation of the steppe zone [62]. There are many Manchurian and even subtropical and tropical Orthoptera.

According to orthopteran distribution, the faunas of the semi-deserts should be included in the Saharan-Gobian subregion [8,9,10,11] (Figure 1(*2*,*6*)). This subregion differs from the Scythian one in the dominance of the subfamilies Thrinchinae and Calliptaminae and the tribes Docistaurini and Sphingonotini. Some species from the tropical tribes Pyrgomophini, Phlaeobini, Eyprenocnemidini, etc., are found in the azonal habitats. There are also some endemic subfamilies and tribes (e.g., Egnatiini) and genera (*Glyphonotus* Redtenbacher, etc.). The prevalence of terricoles is observed on the plains and the hills. The northern part of the subregion includes two provinces associated with the semi-deserts. Both are discussed in this review:

(4) The Kazakhstan Province (Figure 1(*2*)) whose fauna includes Orthoptera associated both with the steppes and the deserts. The endemic species (*Asiotmethis zacharjini*, *Eclipophleps beybienkoi*, *Mesasippus tarbagataicus* Sergeev et Bugrov) are mainly limited to East Kazakhstan. Two districts may be discerned (Central Kazakhstan and Zaisan). The latter is characterized by concentration of endemics and subendemics from the genera *Asiotmethis* Uvarov and *Mesasippus*.
(5) The Mongolian Province (Figure 1(*6*)) is characterized by some subendemics (*Dasyhippus barbipes*, *Bienkoxenus beybienkoi*, etc.). Several districts may be defined (from north-west to south-east: Hirgis-Harausnur, Hangai, Inshan).

The complicated environment of the Eurasian steppes is favorable for many Orthoptera. As a result, there are some centers of Orthoptera diversity and endemism [9]. Several subfamilies, tribes, and generic groups are likely to originate here. Among them are Zichyinae, Onconotini, some groups of Drymadusini and Melanoplinae, the so-called Filchnerellae group of Pamphagidae, Hypernephiini, and Bryodemini. They mainly prefer dry grasslands (plain and mountain steppes and semi-deserts) (Zichyinae, Filchnerellae, Hypernephiini (partly), Bryodemini) or mountain cold meadows (other Hypernephiini). Some other taxa are distributed more or less widely but have endemic genera and species in the region (Platycleidini, Chrysochraontini, Gomphocerini, etc.). Often, they are connected both with the local mountains and with the high plateaus. Other groups are observed near the outer boundaries of the region.

The steppe landscapes of the mountains of Central Asia and Tibet are settled by diverse faunas of Orthoptera. As a rule, they include many species from several unusual and specific groups of these insects, e.g., from Conophymatinae (Conophyminae), Gomphomastacinae [10,11], and also from endemic genera of widely distributed taxa, such as Bergiolini, Drymadusini, and Platycleidini. However, the main local centers of diversity and endemism are in outer (marginal) mountains [63]. For instance, the Tibetan plateau is surrounded by some biogeographic regions with high level of diversity and endemism of Orthoptera [11].

The bush-crickets of the subfamily Zichyinae are mainly Mongolian and Chinese species. Many endemic genera and species of this taxon are distributed over the north-eastern provinces of the Saharan-Gobian subregion. The Phaneropterinae are chiefly associated with subtropical and tropical regions. Some of them are found in the studied area. Most short-winged Odonturini may be characterized as either local endemics or subendemics, especially in the European steppes. The Tettigoniinae katydids are very various. The Drymadusini and the Bergiolini include a number of species with limited distribution. Some of them are the endemics of different mountain ridges. The tribes Gampsocleidini and Decticini are widely distributed over the Palearctic. The Platycleidini distribution is similar to the Drymadusini one. However, as a rule, its species are comparatively widely distributed. It is important that this tribe includes some forms which are associated with the forest-steppe and the steppe life zones (*Montana striata*, *M. tomini,* etc.). There are many mountain endemics as well [10,11]. The only local genus of the Ctenodecticini occurs inside the boundaries of the steppes and the semi-deserts. The subfamily Glyphonotinae is endemic for the central part of the Saharan-Gobian subregion. The very specific tribe Onconotini is distributed over the western parts of the Sarmathian Province. The Conocephalinae katydids are chiefly connected with tropical regions. However, some species of two tribes, namely Conocephalini and Copiphorini, occur in the southern part of the Eurasian steppes.

A few crickets from the family Gryllidae are mainly associated with the southern and the eastern parts of the region. There are also several genera and species of Tridactylidae and Tetrigidae. Many species of all these families prefer habitats of local river and lake valleys.

The Gomphomastacinae (Eumastacidae) is the endemic group for the mountains of Tien Shan, Pamiro-Allay, Qilian (Nan-Shan), Karakoram, the Hindu-Kush, and the Himalayas. Most species have very localized populations which are associated with a definite part of each ridge. The grasshoppers per se (Acridoidea) are the main group of the Palaearctic Orthoptera. The family Pamphagidae includes some common forms. There are several endemic or subendemic genera and species that are associated with the different types of semi-deserts and dry steppes.

The apterous Conophymatinae grasshoppers occur in the mountains of Central Asia including the mountains of Iran, Afghanistan, and the West Himalayas. The Palaearctic Melanoplinae are partly similar to their North American relatives. As a rule, they are short-winged herbicoles, but some species are more or less widely distributed. Calliptaminae are characteristic forms for steppe and semi-desert habitats. They often dominate in the local orthopteran assemblages. Several subfamilies and tribes (Eyprepocnemidinae, Acridinae *s. str.*, Ochrilidini, Phlaeobini, Acrotylini) are chiefly limited by the northern boundaries of the semi-deserts and sometimes penetrate the very dry steppes.

The Chrysochraontini grasshoppers (Gomphocerinae) are limited by the Holarctic boundaries. It includes short-winged species. Some of them are widely distributed. The distribution of the tribe Hypernephiini is very interesting. These short-winged forms occur mainly in the arid mountains of East Kazakhstan and Mongolia. Some genera are in the mountains of Tien Shan, Zagros, the Caucasus, the East Himalayas, and Hengduanshan. Arcypterini, Dociostaurini, Stenobothrini, and Gomphocerini are widely distributed over the Eurasian steppes. Most species of mainly tropical and subtropical Epacromiini, Locustini, and Oedipodini are limited by the southern part of studied area. Bryodemini and Sphingonotini include terricoles that are commonly associated with the semi-deserts and the dry steppes.

The pattern of orthopteran distribution is the result of interaction between both present natural (geographical, ecological, climatic, etc.) conditions and evolutionary history of species colonization. The majority of high taxa consists of tropical and subtropical groups. Only a few subfamilies (Zichyinae, Glyphonotinae, Thrinchinae, Pamphaginae, Conophymatinae) and tribes (Drymadusini, Gampsocleidini, Onconotini, Egnatiini, Chrysochraontini et al.) are mainly associated with the Palaearctic Region. However, almost all taxa have centers of diversity and endemism in the southern part of this area [8,9,10,11]. Some of them are limited by the local mountains.

Relationships between the orthopteran faunas of the Eurasian steppe and the North American prairies are very weak. There is the only common species—*Tetrix subulata* (Linnaeus) [64]—but this form is commonly associated with local flood-plains. Besides that, there are some common genera. However, these genera can be divided into several groups; the first includes genera distributed mainly in North America (*Stethophyma* Fischer), the second one in Eurasia (*Chorthippus*, *Aeropedellus* Hebard), and the third one includes genera (*Conocephalus* Thunberg, *Gryllus* Linnaeus, *Tetrix* Latreille) widely distributed in both temperate and tropical regions.

## 5. Population Distribution: General Patterns

The general pattern of ecological distribution of orthopteran populations over the Eurasian steppes is very complicated. Local populations are distributed over a species range in accordance with natural conditions, especially the Earth’s landscape pattern [65]. These populations can be connected with each other into a single landscape unit and may be divided by different barriers. Completely isolated populations of Orthoptera are exceptional [13]. Such populations are commonly observed only in the cases of non-flying mountain or insular forms or under other specific natural conditions. For instance, the valley and the watershed colonies of the widely distributed grasshopper *Chorthippus parallelus* (Zetterstedt) are quite different over all steppe territories [66]. The significant difference between the local populations on the southern and the northern mountain slopes was described for several grasshopper species in the steppe part of the Altay Mountains. [67].

Distributional analysis of species populations over regions, geographic landscapes, and their units allows for identification of groups with similar ecogeographic relations, including environmental heterogeneity [65]. An analysis of the relationship amongst the different parts of the population system of each species also can show paths of dispersal and potential contacts between local populations.

**Figure 2:**
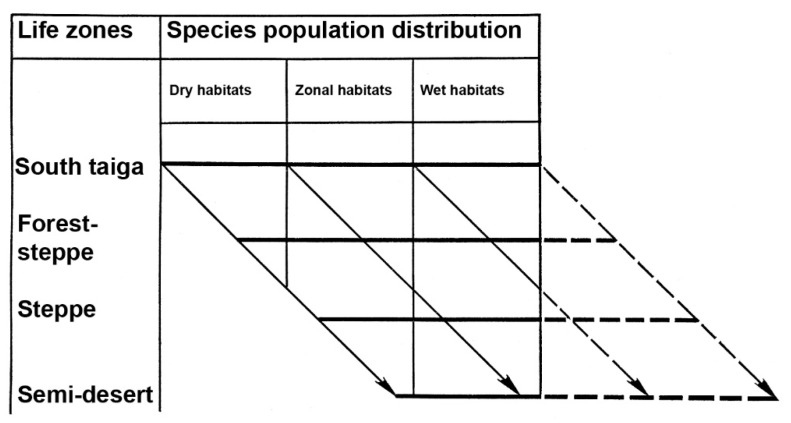
Bey-Bienko’s scheme of the zonal shifting of habitats (after [68]).

The first studies of grasshopper populations distribution were done by G. Ja. Bey-Bienko [68]. He compared the patterns of species distribution from the southern taiga of West Siberian Plain on the north to the semi-deserts of East Kazakhstan on the south along the Irtysh River valley. The analysis of population distributions over the life zones and their habitats, from flood-plains to watersheds, allowed him to reveal the pattern of geographic change of habitats [65,66,68,69,70,71] (Figure 2). For instance, if the species prefers the typical steppe habitats, it commonly colonizes almost all applicable grassland habitats in the steppe life zone. However, in the forest-steppes and in the southern parts of the taiga zone, its populations are associated with some dry habitats (e.g., sandy openings) and are insular and rare. In semi-deserts and deserts, its colonies can occupy the meadows of the local flood-plains and are sporadic and relatively rare. However, such insular populations can be very abundant [68,69,71]. Population distribution may also change not only from north to south but also from west to east [71,72].

The analysis of population distribution within the Eurasian steppes showed that the boundary between the steppe per se and the semi-deserts is significant in demarcating parts of population systems of many species. The frontier between the semi-desert and the northern deserts is also important.

In the Eurasian steppes, a diversity of favorable conditions allows many Orthoptera to coexist at high levels of their abundance. The populations of native Orthoptera extend through all the herbaceous landscapes. Often, their distribution is associated with local watershed plains. Under these conditions, the interrelating of colonies of each species may result in great abundance. In addition, the region is characterized by its widespread species through transformed landscapes [65]. The local types of grasslands are settled chiefly by typical steppe species, while Orthoptera preferring other life zones tend to be found only in insular habitats. Orthopteran populations often overlap and can switch locations because of climatic fluctuations [73]. There are many paths for potential spread of species, including anthropogenic ones [8,70].

Long-term accounts of Orthoptera distribution highly cognate with each other and often have no reliable differences in population distribution of each species [66,67]. However, this commonly seems to be correct for temperate grasshoppers and bush-crickets and solitarious populations of locusts [13,72]. For instance, the analysis of the long-term data confirms that landscape distribution of *Chorthippus parallelus* was not changed significantly for many years [66]. In weakly transformed habitats, its abundance varies but remains approximately at the same level. The species demonstrates constant preference for the steppe meadows of watershed plains. Thereby, landscape distribution of colonies may be considered as comparatively stable for temperate grasshoppers [71,72].

**Figure 3:**
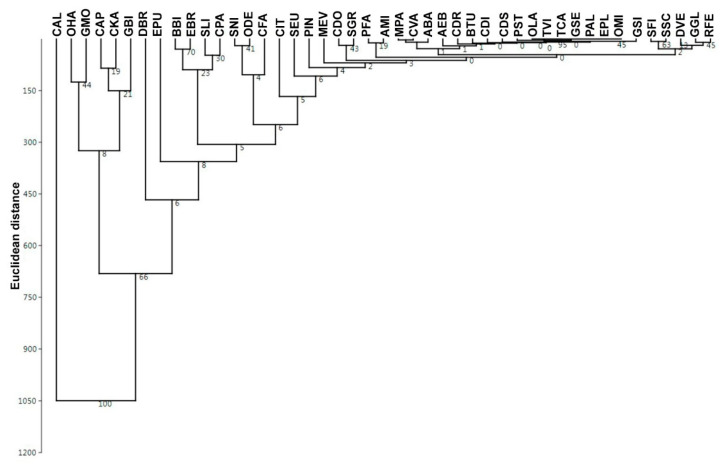
The Euclidean distances between the orthopteran species distributions in the Karasuk River Basin, SE West-Siberian Plain (Ward’s hierarchical clustering) (after original data [74], calculated by PAST 4.02 [75]): digits—supporting percentage of replicates; species: AEB—*Aeropedellus baliolus* Mistshenko; ABA—*Angaracris barabensis* (Pallas); AMI—*Arcyptera microptera* (Fischer de Waldheim); BBI—*Bicolorana bicolor* (Philippi); BTU—*Bryodemella tuberculata* (Fabricius); CTI—*Calliptamus italicus* (Linnaeus); CVA—*Celes variabilis* (Pallas); CAL—*Chorthippus albomarginatus* (De Geer); CAP—*Chorthippus apricarius* (Linnaeus); CDI—*Chorthippus dichrous* (Eversmann); CDS—*Chorthippus dorsatus* (Zetterstedt); CFA—*Chorthippus fallax* (Zubovsky); CKA—*Chorthippus karelini* (Uvarov); CPA—*Chorthippus parallelus* (Zetterstedt); CDR—*Chrysochraon dispar* (Germar); CDO—*Conocephalus dorsalis* (Latreille); CVA—*Celes variabilis* (Pallas); DVE—*Decticus verrucivorus* (Linnaeus); DBR—*Dociostaurus brevicollis* (Eversmann); EPL—*Epacromius pulverulentus* (Fischer de Waldheim); EPU—*Euchorthippus pulvinatus* (Fischer de Waldheim); EBR—*Euthystira brachyptera* (Ocskay); GGL—*Gampsocleis glabra* (Herbst); GSE—*Gampsocleis sedakovii* Fischer de Waldheim; GBI—*Glyptobothrus biguttulus* (Linnaeus); GMO—*Glyptobothrus mollis* (Charpentier); GSI—*Gomphocerus sibiricus* (Linnaeus); MEV—*Montana eversmanni* (Kittary); MPA—*Myrmeleotettix pallidus* (Brunner von Wattenwyl); ODE—*Oedaleus decorus* (Germar); OMI—*Oedipoda miniata* (Pallas); OHA—*Omocestus haemorrhoidalis* (Charpentier); OLA—*Onconotus laxmanni* (Pallas); PFA—*Phaneroptera falcata* (Poda); PAL—*Platycleis albopunctata* (Goeze); PIN—*Poecilimon intermedius* (Fieber); PST—*Psophus stridulus* (Linnaeus); RFE—*Roeseliana fedtschenkoi* (Saussure); SSC—*Stauroderus scalaris* (Fischer de Waldheim); SEU—*Stenobothrus eurasius* Zubovsky; SFI—*Stenobothrus fischeri* (Eversmann); SLI—*Stenobothrus lineatus* (Panzer); SNI—*Stenobothrus nigromaculatus* (Herrich-Schäfer); SGR—*Stethophyma grossum* (Linnaeus); TCA—*Tettigonia caudata* (Charpentier); TVI—*Tettigonia viridissima* Linnaeus.

An analysis of species distribution over the Karasuk River basin crossing the border between the forest-steppe and the steppe in the southern part of West Siberian Plain (see also [74]) shows that three groups of species may be distinguished on the basis of their abundance in the main types of habitats (and when there were no outbreaks!) (Figure 3). The first group includes only *Chorthippus albomarginatus* (De Geer), *s. str*. This species usually prefers the dry meadows with grass dominance. The second group includes several grasshoppers (*Omocestus haemorrhoidalis*, *Glyptobothrus biguttulus* (Linnaeus), *G. mollis* (Charpentier), *Chorthippus apricarius* (Linnaeus), *C. karelini* (Uvarov)) commonly associated with dry meadows and meadow steppes but with variety of forbs and open spaces between plants. All other Orthoptera are in the last group. These species prefer the local steppe habitats and also the wet meadows. A total of 11 species from 45 (*C. albomarginatus*, *C. karelini*, *Glyptobothrus biguttulus*, *Calliptamus italicus* (Linnaeus), *Euchorthippus pulvinatus* etc.) were relatively abundant at least in one habitat studied and occupied some other biotopes as well. Each of the other 13 species was found only in one habitat studied. Among them were *Onconotus laxmanni* (Pallas), *Platycleis albopunctata* (Goeze), *Gomphocerus sibiricus*, and *Stethophyma grossum*.

The comparison of local population distributions of orthopteran species over the small river basins being within the main biogeographic regions allowed us to describe the main trends of their co-existence in the Eurasian steppes [76,77]. Most species are distributed throughout all available habitats of the local river basin at the high levels of abundance. Such forms spread through transformed landscapes. As a rule, the maximum of density of one species does not coincide in space and time with the maximum of others.

**Figure 4:**
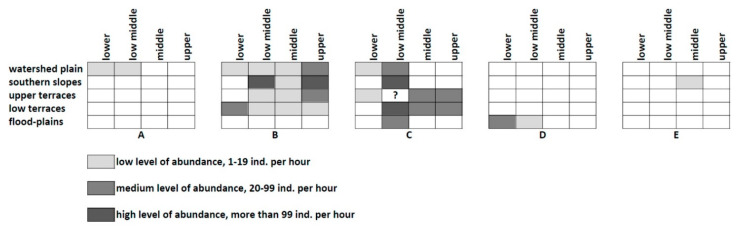
Population distribution of model orthopteran species across the basin of the small Zyrianka River (forest-steppes of SE West-Siberian Plain, SE Novosibirsk). (**A**)—*Psophus stridulus* (Linnaeus); (**B**)—*Chorthippus apricarius* (Linnaeus); (**C**)—*Chorthippus parallelus* (Zetterstedt); (**D**)—*Chorthippus montanus* (Charpentier); (**E**)—*Dociostaurus brevicollis* (Eversmann).

This trend may be revealed for the basin of the small river (Zyrianka) (Figure 4) in the south-eastern vicinities of Novosibirsk (forest-steppes of West Siberian Plain). Several species groups may be distinguished here based of their colonies distribution: (1) species associated with openings on the local watershed plains (three species), commonly very rare (A); (2) widely distributed forms preferring the dry southern slopes of the river valley (six species) with very high abundance (*Glyptobothrus biguttulus*, *Chorthippus apricarius*) (B); (3) species usually colonizing the local terraces with different meadows (also six species); this group includes very abundant *C. dorsatus* (Zetterstedt) and *C. parallelus* (C); (4) species associated with the flood-plains (three species); they are characterized by very low abundance and insular distribution (D); (5) the invasive *Dociostaurus brevicollis* (Eversmann) limited by anthropogenic habitats (E).

**Figure 5:**
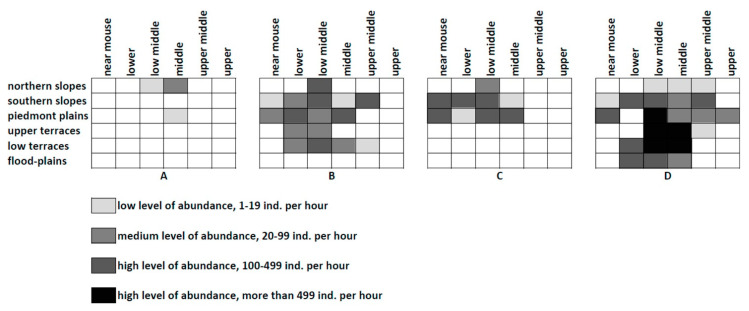
Population distribution of model orthopteran species across the basin of the small Edigan River (mountain steppes of the central part of the Altay Mountains). (**A**)—*Zubovskya koeppeni* (Zubovsky); (**B**)—*Chorthippus hammarstroemi* (Miram); (**C**)—*Stenobothrus eurasius* Zubovsky; (**D**)—*Chorthippus parallelus* (Zetterstedt).

The similar situation was described for the Edigan River in the steppe central part of the Altay Mountains. [77] (Figure 5). The local Orthoptera can be shared between four groups: (1) species associated with the local northern slopes with meadows and openings (six species) (e.g., *Zubovskya koeppeni* (Zubovsky); as a rule, they are relatively rare, and their populations are insular (A); (2) forms preferring the southern slopes with the stony steppes and some bushes (12 species); some can be very abundant (*Chorthippus hammarstroemi*, *Ch. apricarius*, but their distribution across other habitats is usually limited) (B); (3) species associated with the local steppes on the piedmont plain (seven species—*Stenobothrus eurasius*, *Chorthippus fallax* (Zubovsky), etc.); their abundance is relatively low, and their distribution is commonly limited by the steppe habitats (C); (4) species of the river valley (18 species) have the local populations on the flood-plains and the lower terraces; however, some may be very abundant (*Podisma pedestris*, *Stauroderus scalaris* (Fischer de Waldheim), *Chorthippus parallelus*, *C. intermedius* (Bey-Bienko) (D); these species can also be abundant over terraces.

Both biogeographic segregation and penetration of orthopteran species populations were revealed for the central part of the Hungarian Middle Mountain Range [78]. The analysis of species distribution showed that colonies of many local forms overlapped, and the natural and the anthropogenic conditions determined this pattern. Several Pontomediterranean steppe species (e.g., *Omocetus petraeus* (Brisout-Barneville), *Euchorthippus pulvinatus*, *Myrmeleotettix antennatus* (Fieber), *Oedaleus decorus*) penetrated the local mountains and increased diversity.

Spatial and temporal heterogeneity and niche overlapping were also described for grasshoppers in the steppes of Inner Mongolia [79,80]. The analysis of spatial heterogeneity allowed several species groups to be distinguished: (1) xerophilous (*Oedaleus decorus* and *Angaracris barabensis* (Pallas); (2) mesoxerophilous (*Glyptobothrus dubius*, *Myrmeleotettix palpalis* (Zubovsky), *Dasyhippus barbipes*, and *Glyptobothrus* sp. (in the text—*Chorthippus brunneus* auct.) [M.S.]); (3) mesophilous (*Chorthippus fallax*, *Mongolotettix vittatus* (Uvarov)), and (4) hydrophilous (*Chorthippus albomarginatus*, *s. l.*, probably *Chorthippus caliginosus* Mistshenko [M.S.]). Distribution patterns of two species, namely *Calliptamus abbreviatus* Ikonnikov and *Omocestus haemorrhoidalis*, were unique. The three most abundant species (*Dasyhippus barbipes*, *Myrmeleotettix palpalis,* and *Glyptobothrus dubius*) were also separated in time of their active life.

In the steppes, acoustic and visual communication can serve as the very important mechanism for sharing ecological niches [81,82]. As a rule, species with similar habitat and trophic preferences use different acoustic and/or visual signals to avoid possible competition and to keep themselves separate.

## 6. Population Distribution: Locusts

Three species of locusts, namely the Migratory locust (*Locusta migratoria* Linnaeus), the Italian locust (*Calliptamus italicus*), and the Moroccan locust (*Dociostaurus maroccanus* (Thunberg)), occur in the Eurasian steppe. These species have spatially structured populations with rather complicated dynamics. In some parts of their ranges, they are able to change their behavior from solitarious to gregarious and vice versa and irregularly produce devastating upsurges [83,84]. Such populations of these species are not suitable objects for forecasting and modeling [72,85], because in many cases, we do not know well dynamic patterns of the spatial population structures of locusts on the different spatial and temporal scales, especially during recession periods. Biogeographically, a species range and its boundaries should be explored relative to the ecological limits and the general dynamic patterns. Regional studies should include research of spatial population structure distribution inside a region, evaluating barriers and ways for migration, long-term dynamic observations, and genetic and phenotypic trait distribution. At the local level of studies, spatial structures (patches and gaps, distances, population connectivities, barriers, and corridors) and local migration systems associated with a landscape matrix should be revealed.

### 6.1. The Migratory Locust

The Migratory locust is the most widely distributed acridid species [86]. Its range includes almost all the Eurasian steppes. The species is one of the most important transboundary pests in many tropical and subtropical countries of the Old World and of the southern parts of temperate Eurasia as well. However, if in the European steppes and adjacent territories, its populations were and often are very abundant, in the Asian steppes, its colonies were and are very rare and characterized by low densities [87].

In the beginning of the last century, there were areas with very dense populations of the Migratory locust in the forest-steppes and in the southern parts of the deciduous forest life zone of East Europe [88,89,90]: (1) in the Desna River basin; (2) in the middle part of the Oka River basin; (3) near Voronezh on the left side of the Voronezh River; (4) in the Volga River basin near the mouth of the Kama River. These populations were mainly associated with dry and light (sandy or loess) soils [91]. In these conditions, the hopper bands and swarms were very rare, only in very dry years [91]. However, after the 1940s, the situation with this species remained relatively calm.

In contrast with the forest-steppes, the populations of the Migratory locust in the European steppes per se were and are mainly confined by reed beds in the lower parts of the local river basins and around lakes. They remain abundant. During outbreaks, hopper bands and swarms can occupy adjacent areas with agricultural fields and pastures. Z. Waloff [92] discussed history of the so-called West Pontian breeding areas of the Migratory locust relative to its possible northwestward migrations, but the contemporary situation in this territory is also relatively calm. However, the species remains one of the most important pests across territories of South Ukraine and south-east European Russia (from Azov Sea to the Ural Mountains.).

In the first half of the 20th century, adults of the Migratory locust were found in many places in the southern part of West Siberian Plain (up to the southern taiga). However, these specimens mainly originated from South-East Kazakhstan [39], where some permanent habitat areas of this species were and are in the northern deserts (near Zaisan, Balkhash, Shalkar, Alakol lakes) where its upsurges commonly start. However, at least several stable populations were known in the south-eastern part of the Plain. They could be recognized by common presence of larvae and young undamaged adults [28]. In recent years, arrivals of swarms became very rare, but at least several populations of the Migratory locust exist in the region [87,93]. They are mainly associated with the typical habitats of the species, i.e., reed beds.

In 2017, a moderate population of the Migratory locust was found in Tuva (the Republic of Tyva) in the Altay-Sayan Mountains, near hypersaline Shara Lake. The discovered population inhabited several adjacent habitats: the local dry meadows with dominance of *Achnatherum splendens* (Trin.) Nevski on the lake terraces, some meadows with short mesophilous grasses along a small stream, and halophytic meadows in wide depressions near the lake. The Migratory locust was very abundant on the lake terraces; its density was up 1 ind. per m^2^. The locust aggregations were not observed both for adults and hoppers. The main traits were typical for the solitarious form, however, some traits of adults indicated that the population could be characterized by some level of gregarization [87]. The ratios of posterior femur length to maximum width of head and pronotum length to maximum width of head overlapped partly with the values typical for the gregarious forms of the Migratory locust [94]. The populations of this species are also very rare and sporadic over the steppes of East Siberia and Mongolia.

### 6.2. The Italian Locust

The Italian locust is a common species and the most important pest in the Eurasian steppes from their western limits up to the south-eastern parts of West Siberian Plain and East Kazakhstan. Numerous outbreaks of this species were described for these territories [44,83,95,96]. The first data on its population distribution in the European steppes were published by K. Lindemann [97]. Later, I. A. Chetyrkina [98] analyzed the patterns of species population distribution in the semi-deserts of East Kazakhstan during the recession years, and K. A. Vasil’ev [99] described in details the spatial distribution patterns of bands and swarms of the Italian locust in the dry steppes and the semi-deserts of Central Kazakhstan.

Recently, the main data on the Italian locust distribution and ecology were summarized for the territory of the former USSR. The main part of the species range is connected with the semi-desert zone [85,100,101]. There is its optimum, where many outbreaks have been often observed. As a rule, species abundance is great. In any event, as judged by available data, the Italian locust renders a preference of landscapes with the mosaic xeric plant cover.

In the forest-steppes of West Siberia, the Italian locust is widely distributed. However, its populations are very localized and are at a low level of abundance across the overgrazed plain pastures. In the steppes, its populations occupy not only local watershed plains but the dry parts of upper flood plains and lower terraces and the stony southern slopes of hills. In this area, the specific additional optimum of this species is observed in the dry steppes of Kulunda [85]. This optimum partly covers a region of the dry pine forests of the Irtysh region. Often, outbreaks occur here [102,103]. The presence of two optima near the east boundaries of Kazakhstan—main and additional—corresponds to the picture described by Stolyarov [104] for West Kazakhstan. Both optima seem to stretch from west to east through the whole eastern part of the Italian locust range as a tape [100].

Spatial population distribution of the Italian Locust at periods between outbreaks both within the whole area and on better examining territory of the eastern parts of it range corresponds as a whole with the known rule of habitat change [68]. In the northern part, the species is distributed over very dry habitats. In the central one, the species prefers comparatively xeric and varied habitats of the steppe and the semi-desert life zones. However, in the southern part, its colonies are usually localized either in mesohygrophytic habitats of river valleys or in mountains.

It is important to recognize that the population dynamics within additionally infested areas differs significantly from those in the main area [85] because they are situated in different climatic zones. Marginal areas of high abundance and, probably colonies as well, can occupy different ecosystems, even in one small region (e.g., flood plains and watershed plain) [85]. The analysis of the long-term dynamics of several local populations of the Italian locust in the north-eastern part of its range showed that they were characterized by quite different variations [105]. For instance, these populations can be distinguished by the years of maximal development of the outbreak [105]. The similar pattern was later revealed for the North Caucasus as well [106].

### 6.3. The Moroccan Locust

The Moroccan locust occurs only in the European steppes and, furthermore, it is distributed over the steppes of the Crimean Peninsula and the North Caucasus [107]. However, the insular part of its range is in Pannonian Plain [108,109]. Its populations were and are sporadically distributed over the southern steppes of South-East Europe [110]. However, in this region, serious outbreaks were registered in the end of the 19th and in the beginning of the 20th century [110]. They were mainly associated with the overgrazed dry steppes of south Crimean Peninsula and plains (including the piedmont ones) of the North Caucasus between Azov and Caspian seas. In the beginning of the 20th century, some dispersed populations of the Moroccan locust were also observed in the southern parts of Ukraine [110]. In almost all areas, this species prefers the short dry grasslands with dominance of *Poa bulbosa* Linnaeus and on undisturbed clay soils [111]. Intensive transformation of the dry steppes into agricultural fields often resulted in either significant reduction of the species abundance or elimination of its populations [111]. In the end of the 20th century, A. V. Latchininsky [111] noted that the probability of the species upsurges in the North Caucasus and the Crimean Peninsula was extremely low. Our unpublished data on orthopteran distribution over the mountain steppes of the East Caucasus supported this idea. However, after 2011, serious outbreaks of the Moroccan locust started again in the dry steppes of the North Caucasus and the Crimean Peninsula. The similar situation was described for its population in Pannonian Plain, where, after several calm decades, the restricted upsurge developed in 1993 [109,112]. In all cases, the significant changes of the population distribution and the dynamics were mainly determined by changes of human activity in local landscapes [110,111,112].

## 7. Population Distribution: Rare Species

The fauna of the Eurasian steppes includes a few rare species. There are several implicit areas of Orthoptera diversity [113,114]. These weak acridid foci of diversity often coincide or overlap the areas of locust and grasshopper upsurges [65,113,114]. As a result, some serious problems related to population management of both abundant and rare species may arise [65,115]. The ranges of some species are limited; usually, such species are endemics or subendemics of a biogeographic province. Populations of other orthopterans suffered from human activity, because huge areas of the steppes were ploughed or overgrazed. Local colonies of rare species may be extremely different. Some local colonies of rare species are critically endangered or under threat of extinction [114]. Some colonies of rare grasshoppers are very locally abundant for long periods, while a few are continuously stable but at a low level of abundance. Many rare species are brachypterous or wingless, especially in the mountains of the region [116]. Besides, some local colonies of pest species (e.g., *Calliptamus italicus* or *Dociostaurus maroccanus*) may be also almost extinct, but others are abundant [72,111].

About 1500 orthopteran species are now on the International Union for Conservation of Nature (IUCN) Red List of Threatened Species [117], and many are on regional Red Lists/Books, etc. [118]. The actual version of the International Red List includes 26 species which can be associated with the Eurasian steppes. However, almost all species occur in the European parts of the region. Several species are critically endangered (CR), including *Isophya boldyrevi* Miram, *I. doneciana*, *Bradyporus multituberculatus montadoni* (Burr) (as a separate species—M.S.). Listed as endangered (EN) are *Isophya stepposa*, *I. zubovskii* Bey-Bienko, *Bradyporus macrogaster* (Lefebvre), and *Asiotmethis tauricus*. Some forms (including widely distributed *Saga pedo* and *Stenobothrus eurasius)* are vulnerable (VU). This list does not include one of the rarest taxa of the European steppes—*Bradyporus multituberculatus multituberculatus*. The species is on the Red Books of Russia and Ukraine [118]. It was relatively widely distributed over almost all steppes of South-East Europe and was more or less abundant in virgin steppe landscapes until the middle of the last century, but their populations became nearly extinct during its second half [118,119]. Fortunately, at least one population of this bush-cricket was found recently in Kabardino-Balkar Republic (Russia) [119].

Unfortunately, the actual version of the IUCN Red List of Threatened Species does not include the steppe species associated with the steppes of South Siberia, Kazakhstan, Mongolia, and North China or with the steppe mountain landscapes of mountains of Central Asia. There are only some widely distributed species (e.g., *Saga pedo*, *Gampsocleis glabra* (Herbst), *Onconotus servillei* (Fischer de Waldheim)). However, some endemic and subendemic genera, species, and subspecies occur in the central and the eastern parts of the Eurasian steppes. Some of them are known from one or several localities. Besides, populations of many orthopteran insects can decline in regions with intensive agriculture.

Several rare species occur in a few natural reserves in the Eurasian steppes, for instance, in the “Bugeac” Natural Reserve in Moldova [120], the Biosphere Reserve Askania-Nova in South Ukraine [23], the Central Black Earth Biosphere Reserve (Kursk Region, Russia) [121,122], the Belogorye Reserve (Belgorod Region, Russia) [122], the Bogdo-Baskunchak Reserve (Astrakhan Region, Russia) [81], and the Ubsunur Hollow Biosphere Reserve (Tyva Republic, Russia) [47,48,49].

Recently, distribution patterns of rare acridid species in the south-eastern part of West Siberian Plain were analyzed [93]. The ranges of two species are limited by the Kulunda steppe and the adjacent semi-deserts of East Kazakhstan (*Asiotmethis jubatus* and *Mesasippus arenosus*). The distribution patterns of several species, e.g., *Asiotmethis muricatus* (Pallas), *Notostaurus albicornis* (Eversmann), and *Mesasippus arenosus,* did not change significantly during the last decades. Two species, namely *Megaulacobothrus aethalinus* and *Aeropedellus variegatus*, were recently found near the boundaries of the region. Four species (*Asiotmethis jubatus*, *Arcyptera fusca* (Pallas), *Stenobothrus carbonarius* (Eversmann), *Sphingonotus coerulipes*) are extremely rare now. The local populations of almost all rare species are usually associated with a few types of habitats. This means that intensification and changes of human activity may result in their extinction.

## 8. Conclusions: The Steppe Orthoptera—Retrospective and Prospective

Reconstruction of the past of many taxa and faunas is problematic. The most important problem is the shortage of appropriate fossils. This results in development of different hypotheses explaining geographic and ecological history of different taxa and faunas. In the absence of adequate fossil data, some possible approaches can be based on a complex analysis of the ecological factors, adaptations to particular living conditions, and the optimum conditions, which can be estimated by some comparative studies of species range shapes and the population distribution within the range [8,60,70].

The history of Orthoptera of the Eurasian steppes was discussed in a number of papers. A century ago, Uvarov [21,123] and D. P. Dovnar-Zapolsky [22] emphasized strong relationships between the European steppe fauna and the Mediterranean faunas. Uvarov [123] also noted that all European faunas suffered extremely during last glaciations. He also noted that the European steppe fauna showed evident affinities with the Siberian one and supposed that many orthopteran taxa migrated to Europe from Asia. Uvarov suggested that some specific group of Orthoptera had been originated in the eastern territory of temperate Asia. He called this group “the Angara fauna” (or Angarian fauna) and included in it the group Chorthippi (i.e., *Chorthippus* and its relatives), the genera *Podisma* Berthold, *Stethophyma*, *Podismopsis* Zubovsky, etc. He also mentioned some relationships between the Angarian fauna and the faunas of the southern parts of East Asia. Bey-Bienko [12] tried to develop some of Uvarov’s ideas further. He also wrote about the evident association between the European steppe and the Mediterranean faunas of Orthoptera. However, some species of the European steppes (especially the forest-steppes) are related to the European meadow-forest fauna. The significance of all western faunas declines in the central and especially in the eastern parts of the steppes. The last ones are strongly connected to the faunas of East Asia. In the southern steppes and the semi-deserts of south West Siberia and Kazakhstan, there are many species mainly distributed in the arid regions of Central Asia. The orthopteran faunas of East Asia and Mongolia are linked to the faunas of Gobi Desert. Bey-Bienko also emphasized the relatively young age of the steppe faunas but did not try to further evaluate it. Later, Sergeev [8,65,124] hypothesized that all Eurasian faunas associated with forest-steppes, steppes, and semi-deserts could have separated mainly in the end of Pliocene, but their modern diversity and distribution could have been determined by events of the last millenia (see also [125]).

One of the principal results of retrospective views on faunas and populations is the opportunity to forecast their possible changes in the future. If the trend of global warming continues, the steppe zone will shift northwards [126], and the effect combined with continuing strong anthropogenic pressure will result in significant changes to the Orthoptera distribution pattern. Local endemics and subendemics may be eliminated due to high rates of change, while local populations of many widely distributed forms decline. The overall effects may be particularly significant for insular mountain populations, because their native landscapes may disappear.

The steppe species will shift the northern boundaries of their ranges northwards, and some of them will be able to use numerous transformed habitats, such as clearings, roadsides, agricultural fields, and pastures [38]. Besides that, their abundance may increase, and some of them may become potential pests. For instance, in South Siberia, the Migratory locust will able to colonize some new and unusual types of habitats. The local populations may become very abundant and gregarious, at least to some extent [87]. It is likely that there will be an increase both in the abundance of the Migratory locust and in the presence of more local populations, which may result in serious problems for plant protection.

In Hungary, B. Nagy [78] noted significant decreasing of populations of several species, including endemic *Isophya costata* and also *Saga pedo*, *Arcyptera fusca*, *A. microptera* (Fischer de Waldheim), and *Stauroderus scalaris*, due to human activity

In the northern steppes of Central Russian upland, A. V. Prisny [122] showed possible extinction of almost all populations of several grasshopper species, namely *Arcyptera fusca*, *A. microptera*, *Stenobothrus fischeri*, *Bryodemella tuberculata*, *Psophus stridulus* Linnaeus, and *Sphingonotus caerulans* Linnaeus. He explained this declining by intensification and some changes of human activity. Besides, the isolated colony of *Sphingonotus coerulipes* was unexpectedly found on stockpiles of mining plants.

Significant changes of orthopteran diversity were revealed for the vicinities of Lake Baskunchak in the semi-deserts of South-East Europe [82]. Several species, namely *Podisma pedestris*, *Euthystira brachyptera* (Osckay), *Gomphocerus sibiricus*, *Psophus stridulus*, *Bryodemella tuberculata,* etc., were found here in the middle of the 19th century [127] but disappeared until the beginning of the last century. V. Yu. Savitsky [82] supposed that these shifts were determined by climatic changes. On the contrary, in the south-eastern part of European Russia, the range boundaries of some species mainly associated with the desert life zone shifted northwestwards (*Dericorys tibialis* (Pallas), *Heteracris adspersa* (Redtenbacher), *Sphingonotus halophilus* Bey-Bienko, *Ramburiella turcomana* (Fischer de Waldheim), etc.) [128]. The bush-cricket *Glyphonotus thoracicus* (Fischer de Waldheim) mainly associated with river valleys of deserts and semi-deserts was recently found in the southern parts of the steppes of Central and North-East Kazakhstan [129].

Our published and unpublished data show conspicuous changes in distribution of several species in the south-eastern part of West Siberian Plain during the last decades. Populations in the first half of the 20th century of the widely distributed and abundant *Gomphocerus sibiricus* significantly declined in the plain steppe habitats during the last decades [74]. Several other species (e.g., *Podisma pedestris*, *Sphingonotus coerulipes*) became very rare [93,114]. However, the northern and the north-eastern range boundaries of some species shifted mainly northeastwards. Some of them moved over kilometers (*Arcyptera microptera*), some others crossed more than 100 km (for instance, *Phaneroptera falcata* (Poda), *Conocephalus fuscus* (Fabricius), *Dociostaurus brevicollis*, *Oedipoda caerulescens* (Linnaeus), *Oedaleus decorus*, *Epacromius pulverulentus*) [70,114]. Almost all species actively colonize transformed habitats such as lawns, roadsides, and agricultural fields [114]. Similar combination of some positive and negative trends in orthopteran diversity and distribution changes mainly associated with local human activity is also revealed for Central European grasslands [130,131] and the mountains of Switzerland [132]. On the contrary, our unpublished data show that the distribution of orthopteran taxa and populations over the steppe intermountain basins of the Altay-Sayan Mountains remains relatively stable.

I can conclude that, during last decades, biological diversity, geographic, and ecological distribution of Orthoptera over the Eurasian steppes have been characterized by rapid changes due to climatic variations and changes of local human activity. In many cases, we can observe declining and elimination of local populations but, on the contrary, other species can colonize new areas and habitats. This is true for both possible pests and rare species. This means we should constantly monitor orthopteran populations and assemblages over all huge territory occupied by the steppe biome.

## Figures and Tables

**Table 1:** Estimation of species richness in different parts of the Eurasian steppes.

Life Zone	According Bey-Bienko [12] for the Steppe Territory of the Former USSR	Actual Estimations	
		West Siberia—Kazakhstan	East Siberia—Mongolia
Forest-steppe	90	74	98
Steppe	150	105	114
Semi-desert	120	138	105
